# Association between tooth loss and hypertension among older Chinese adults: a community-based study

**DOI:** 10.1186/s12903-019-0966-3

**Published:** 2019-12-09

**Authors:** Dongxin Da, Fei Wang, Hao Zhang, Xiaoli Zeng, Yiwei Jiang, Qianhua Zhao, Jianfeng Luo, Ding Ding, Ying Zhang, Bei Wu, and the Shanghai Aging Study

**Affiliations:** 1 Department of Preventive Dentistry, Shanghai Stomatological Hospital, Fudan University, 356East Beijing Rd, Shanghai, 200001 China; 2 Oral Biomedical Engineering Laboratory, Shanghai Stomatological Hospital, Fudan University, Shanghai, China; 3 Department of Biostatistics, School of Public Health, Fudan University, Shanghai, China; 4 Institute of Neurology, Huashan Hospital, Fudan University, Shanghai, China; 5 Rory Meyers College of Nursing and NYU Aging Incubator, New York University, 433 First Avenue, New York, NY 10010 USA; 6 NYU Aging Incubator, New York University, New York, NY USA

**Keywords:** Teeth, Hypertension, Community, Older adults

## Abstract

**Background:**

The purpose of the study is to examine the association between tooth loss and hypertension among older community residents in urban China.

**Methods:**

This study included 3677 participants aged ≥50 years from the Shanghai Aging Study. We determined the number of teeth missing from questionnaires. Hypertension was defined as the mean of two measurements of systolic blood pressure (SBP) (140 mmHg or higher), diastolic blood pressure (DBP) (90 mmHg or higher) or physician-diagnosed hypertension confirmed from medical records. A multivariable logistic regression model was used to investigate the association between tooth loss and hypertension.

**Results:**

The average number of missing teeth among study participants was 9.67. Among them, participants with hypertension had lost an average of 10.88 teeth, significantly higher than those without hypertension (8.95) (*p* < 0.0001). After adjusting for covariates (socio-demographic characteristics, health behaviors and other chronic conditions), teeth lost (15 or more) was significantly associated with grade III hypertension, with OR = 1.55(95% CI 1.09–2.20).

**Conclusions:**

Significant tooth loss maybe associated with severe hypertension among older Chinese adults. Prevention of tooth loss is important to the overall health of this population.

## Background

Hypertension is considered the leading risk factor for life-threatening diseases [1–4].Approximately 54% of stroke and 47% of ischemic heart disease cases have increased blood pressure [5].According to a national survey of cardiovascular disease conducted in China in 2015, the prevalence of hypertension increased from 2002 (17.7%) to 2012 (25.2%). In 2010, hypertension caused approximately 2 million deaths in China [6].

The number of remaining teeth is an important indicator for oral health [7]. According to the 2005 Chinese National Oral Health Survey, 4.5% of elderly Chinese were edentulous and those aged 65–74 years had lost an average of 10 teeth [8]. Numerous studies have shown that periodontitis and tooth loss caused by oral inflammation is significantly associated with hypertension, excluding other risk factors [4]. Numerous studies on tooth loss and hypertension have been carried out around the world. These studies found a significant association between hypertension or increased blood pressure and periodontal disease or tooth loss [9–21].

Studies conducted in high income countries showed a great variation in study design, sample sources and data analyses in this topic area. No studies have been conducted among the Chinese population. Due to differences in health care system, culture, genetic factors and health literacy, it is unclear whether the evidence generated from other countries is generalizable to the Chinese population. For example, people in western countries consume more high-cholesterol foods than Chinese people. The prevalence of hypertension among Americans is 32% but is 25.2% in China [6, 22].Therefore studies on older Chinese people are necessary. In addition, the prevalence of hypertension and cardiovascular disease is rapidly rising in China. Identifying risk factors for hypertension and cardiovascular disease has important clinical and policy implications for healthcare professionals and policy makers to develop targeted interventions and programs to prevent and treat hypertension. To address the knowledge gap in the literature, this study aimed to examine the association between tooth loss and hypertension among older community residents in urban Shanghai, China.

## Methods

### Study participants

All participants were recruited from the Shanghai Aging Study (SAS). The SAS aimed to investigate the prevalence of dementia and mild cognitive impairment among older adults living in Jingansi community, Shanghai [23]. Between January 2010 and December 2012, 3836 residents aged 50 years or older were enrolled by SAS, excluding any living in nursing homes or other institutions. Detailed inclusion and exclusion criteria were published elsewhere [23]. Finally, 3677 out of 3836 participants with complete and reasonable self-reported tooth loss were selected in the current study.

### Blood pressure measurement and definition of hypertension

In a seated position, blood pressure was measured twice after at least 5 min of rest using a digital electronic tensiometer (M4; OMRON Corp., Kyoto, Japan); a proper cuff for the patient’s left arm at heart level was used [24]. Hypertension was defined as the mean of two measurements of systolic blood pressure (SBP) (140 mmHg or higher), diastolic blood pressure (DBP) (90 mmHg or higher) or physician-diagnosed hypertension confirmed from their medical records [1, 25, 26]. According to the 2010 Chinese guidelines for the management of hypertension, stage III hypertension was defined as the mean of two measurements of SBP (180 mmHg or higher) or DBP (110 mmHg or higher), stage II hypertension as the mean of two measurements of SBP (160–179 mmHg) or DBP (100–109 mmHg), stage I hypertension as the mean of two measurements of SBP (140–159 mmHg) or DBP (90–99 mmHg) and normal blood pressure as the mean of two measurements of SBP (less than 140 mmHg) or DBP (less than 90 mmHg) [26, 27].

### Observation of tooth loss

Participants in the study received a self-administered oral health questionnaire (Additional file 1) and answered questions about their numbers of missing teeth by themselves. The interviewers counted the number of missing teeth (including third molars) and reviewed the questionnaires. After analyzing the data distribution, we categorized the participants into three groups based on tertiles of the data distribution of tooth loss:(1) 0–3 missing; (2) 4–14 missing; and (3) ≥15 missing.

### Data collection of other variables

Trained research nurses and physicians interviewed the participants face-to-face in order to obtain all relevant information including demographic variables and participants’ socioeconomic status. Demographic variables included age, sex, body mass index (BMI) and number of formal years of education. The BMI was calculated according to the following formula: *person’s weight in kilograms/height in meters squared.* Obesity was defined as BMI ≥27.5 kg/m^3^ based on WHO definition for Asian populations was defined as BMI ≥27.5 kg/m^3^ based on WHO definition for Asian populations. Detailed definitions of variables mentioned above have been published elsewhere [23]. Finally, medical histories were collected by self-report and confirmed with participants’ medical records, and consisted of diabetes and heart disease (including coronary artery disease and arrhythmia).

### Statistical analysis

For the current data, rates of participants with ≥15 tooth lost in hypertension group and non-hypertension group were 0.268 and 0.192, due to the sample size of 3677, the power of test (1 − β) exceeded 95% with α = 0.05, according to the below formulation. Therefore the sample size was sufficient in our study.

$$ n=\frac{Z_{\alpha /2}\sqrt{2\mathrm{p}\left(1-\mathrm{p}\right)}+{Z}_{\beta}\sqrt{{\mathrm{p}}_0\left(1-{\mathrm{p}}_0\right)+{\mathrm{p}}_1\left(1-{\mathrm{p}}_1\right)}}{{\left({\mathrm{p}}_1-{\mathrm{p}}_0\right)}^2} $$

Continuous variables were described in mean and standard deviation (SD), and frequencies (%) were used for categorical variables. We used the Student t-test, Pearson Chi-squared test, analysis of variance (ANOVA) and Cochran–Mantel–Haenszel Chi-squared to compare the variables. The association between the number of teeth missing and hypertension was examined by three ordinal logistic regression models. Odds ratio (OR) and 95% confidence intervals (CI) presented the measurement of the association. Model 1 was a univariate model and Models 2 and 3 were multivariable models. Model 2 was adjusted for age and sex; Model 3 was further adjusted for confounders such as age, sex, socioeconomic factors (education and household income), health compromising behaviours (smoking and drinking), Obesity and medical history (heart disease and diabetes). The group of people with≤3 teeth lost was the reference category for all models.

The *p*-values and 95% CIs were estimated in a two-tailed manner, and *p* < 0.05 was considered significant. Data were analyzed using SAS 9.3 (SAS Institute Inc., Cary, NC, USA).

## Results

The current study included 3677 community-dwelling older adults, aged60 years or above who responded the oral health questionnaire, and received blood pressure measurement. Among them, 1644(44.71%) were men. The mean age was 70.23(SD 8.61) years and mean years of education was 11.69(SD 4.08) years. Among all the participants, 1339 were diagnosed with hypertension. Significant differences in age, BMI, cigarette smoking, heart disease, diabetes, dental caries, being obesity and tooth loss were found between participants with hypertension and non-hypertension. Participants with hypertension were older (mean 72.72,SD 8.62 years),had fewer years of education(mean 11.41,SD 4.49)and had higher prevalence of heart disease (19.10%) and diabetes(19.24%).Participants with hypertension had significantly more teeth missing (mean 10.88) than those without hypertension (mean 8.95) (*p* < 0.001) (Table 1). More than one-quarter of participants with hypertension had lost ≥15 teeth, which was higher compared to non-hypertension participants. Also, the number of teeth missing was associated with hypertension. Subjects with ≥15 teeth lost reported higher prevalence of hypertension (*p* < 0.001) (Table 1).

**Table 1:** Demographic, social economic and medical history of the participants with hypertension and non-hypertension

Variates	ALL *N* = 3677	Tooth loss≤3 N_1_ = 1574	Tooth loss 4–14 N_2_ = 1296	Tooth loss≥15 N_3_ = 807	*P* value
Sex					0.0002
Male, n(%)	1644 (44.71)	644 (40.91)	606 (46.76)	394 (48.82)	
Female, n(%)	2033 (55.29)	930 (59.09)	690 (53.24)	413 (51.18)	
Age,years, mean(SD)	70.23 ± 8.61	67.51 ± 7.99	69.92 ± 7.65	76.06 ± 8.41	<.0001
Education,years, mean(SD)	11.69 ± 4.08	11.81 ± 3.87	11.89 ± 3.86	11.12 ± 4.74	0.0229
Body mass index, mean(SD)	24.26 ± 3.53	24.14 ± 3.33	24.55 ± 3.47	24.03 ± 3.96	0.0002
Monthly household income, *n* (%)					0.0039
< 500RMB	37 (1.01)	11 (0.71)	13 (1.01)	13 (1.62)	
500-1200RMB	52 (1.42)	15 (0.96)	16 (1.24)	21 (2.62)	
> 1200RMB	3564 (97.56)	1534 (98.33)	1262 (97.75)	768 (95.76)	
Cigarette smoking, *n*(%)	390 (10.67)	131 (8.39)	158 (12.22)	101 (12.61)	0.0006
Alcohol drinking, *n* (%)	298 (8.24%)	122 (7.87)	103 (7.98)	73 (9.13)	0.5434
Heart disease, *n*(%)	443 (12.13)	142 (9.13)	146 (111.28)	155 (19.30)	<.0001
Diabetes	498 (13.62)	180 (11.58)	182 (14.05)	136 (16.85)	0.0016
ADL, mean(SD)	21.01 ± 5.01	20.71 ± 4.68	20.77 ± 4.30	21.97 ± 6.40	<.0001
Obesity, n(%)	1435 (38.87)	579 (36.79)	552 (42.59)	304 (37.67)	0.0044
Hypertension	1908 (51.89)	749 (48.14)	688 (53.09)	471 (58.36)	<.0001
Stage of hypertension					<.0001
Non-hypertension	1676 (45.58)	820 (52.10)	569 (43.90)	287 (35.56)	
I	1179 (32.06)	457 (29.03)	446 (34.41)	276 (34.20)	
II	544 (14.79)	208 (13.21)	199 (15.35)	137 (16.98)	
III	278 (7.56)	89 (5.65)	82 (6.33)	107 (13.26)	

The ORs for number of teeth missing among participants with or without hypertension are shown in Table 2.The univariate Model 1 showed that participants who lost ≥15 teeth had an OR for hypertension of 1.97(95% CI 1.65–2.35), and OR for grade III hypertension of 3.44(95% CI 2.52–4.69).After adjustment for sex and age, ≥15 tooth lost was associated with a 21% (OR = 1.21, 95% CI 1.00–1.47) higher odds of hypertension (Model 2). Model 3 showed that tooth loss of ≥15 was significantly associated with grade III hypertension with OR = 1.55(95% CI 1.09–2.20).

**Table 2:** *OR* (95% CI) for hypertension and different stages of hypertension by categories of tooth loss

	Model 1 OR(95% CI)			Model 2 OR(95% CI)			Model 3 OR(95% CI)		
	Tooth loss≤3	Tooth loss 4–14	Tooth loss≥15	Tooth loss≤3	Tooth loss 4–14	Tooth loss≥15	Tooth loss≤3	Tooth loss 4–14	Tooth loss≥15
Hypertension	1.00(reference)	1.39 (1.20–1.61)	1.97 (1.65–2.35)	1.00(reference)	1.18 (1.01–1.38)	1.21 (1.00–1.47)	1.00(Reference)	1.14 (0.97–1.34)	1.18 (0.96–1.44)
Stage of hypertension									
I	1.00(Reference)	1.41 (1.19–1.67)	1.73 (1.41–2.11)	1.00(Reference)	1.26 (1.06–1.50)	1.27 (1.02–1.57)	1.00(Reference)	1.22 (1.02–1.45)	1.23 (0.98–1.54)
II	1.00(Reference)	1.38 (1.10–1.72)	1.88 (1.46–2.43)	1.00(Reference)	1.08 (0.86–1.36)	0.91 (0.68–1.19)	1.00(Reference)	1.06 (0.83–1.34)	0.92 (0.69–1.23)
III	1.00(Reference)	1.33 (0.97–1.83)	3.44 (2.52–4.69)	1.00(Reference)	1.06 (0.76–1.46)	1.63 (1.16–2.29)	1.00(Reference)	0.97 (0.69–1.35)	1.55 (1.09–2.20)

Note:stage **I**: 140 ≤ *SBP* < 160 or 90 ≤ *DBP* < 100; **II**: 160 ≤ *SBP* < 180 or 100 ≤ *DBP* < 110; **III**: 180 ≤ *SBP* or 100 ≤ *DBP*; Model 1:univariate;Model 2: adjusted for sex and age**;** Model 3: adjusted for age, sex, education duration, cigarette smoking, alcohol drinking, heart disease, diabetes, and obesity

## Discussion

This cross-sectional study indicated a positive association between tooth loss and hypertension in community-dwelling older adults in Shanghai, China. Loss of more than 15 teeth was positively associated with hypertension after adjusting for covariates.

Consistent with other observational studies, a strong association between tooth loss and hypertension was observed after controlling for a number of confounding variables. Some studies have reported that tooth loss is associated with higher SBP [14–16, 28, 29] and peripheral arterial disease among men [30].A cross-sectional study in Indian adults indicated that participants with partial tooth loss had 1.62 times (95% CI1.12–2.35) higher OR of developing hypertension after adjusting for all confounders, compared to those with no tooth loss [4].Moreover, the cross-sectional study of Peres et al. suggested that edentulous people have an 8.3 mmHg(95% CI 0.1–16.7) higher SBP compared to individuals with more than 10 teeth in both arches after adjustment [13].The significant association between missing teeth (> 10 missing) and hypertension was also observed among a subset of < 65 years old in a French cohort study with OR = 1.17 (95% CI 1.04–1.31) [31]. Ayo-Yusuf found in 2008 that total tooth loss was significantly associated with hypertension in South African adults, with OR = 1.35(95% CI 1.02–1.78) [9].Additionally, after adjusting for obesity, hypercholesterolemia and hypertriglyceridemia, Taguchi et al. demonstrated that the OR of having hypertension in postmenopausal women with missing teeth was 3.59 (95% CI 1.10–11.7) [15].

Several mechanisms have been proposed to explain the association between tooth loss and hypertension. Due to masticatory insufficiency, tooth loss may alter the eating habits of individuals and so cause less intake of vitamins, fiber and more cholesterol, consequently increasing risk of hypertension [11, 29, 32].Since China is traditionally an agricultural country, high-fiber food still constitutes most of the diet of the study participants. Subsequently, partial tooth loss might be the cause of reduced fiber intake, which is considered to be one risk factor for hypertension [4]. Other potential mechanisms include chronic periodontal inflammation caused by periodontal pathogens, which produce chronic systemic inflammation and vasculopathy and increase the risk of hypertension and cardiovascular diseases in those with periodontal disease [30, 33, 34]. This was further verified by a study which demonstrated a significant association between periodontal pockets and hypertension [17] but no association was found between hypertension and number of teeth [10].

Self-reported number of teeth has been proved effective and is widely used in observational studies. Previous studies conducted in the United States [34] and Japan [33] suggested no significant difference between self-reported number of teeth and the numbers from clinical examination data. In our study, the participants had an average of 9.67 missing teeth, comparable to the findings from large national oral health surveys using clinical examination data in China, in which adults aged 60–74 years had an average of 10 missing teeth [8]. Furthermore, to avoid recall bias, the interviewers observed, counted and confirmed the number of teeth missing for each participant [35], therefore we collected relatively accurate numbers for tooth loss in this study.

Tooth loss is an oral health problem accumulated across life span, and worsens in later life. Severe periodontal disease, unhealthy diet and poor dental care can lead to tooth loss. Given that tooth loss and hypertension are both common among older adults, it is possible that the association between hypertension and tooth loss is bidirectional among those with significant tooth loss [35](those who lost ≥15 teeth in our study).

The strengths of our study are the relatively large sample size for a study of such scale. We adjusted for relevant confounders sequentially in our three models. However, the study has certain limitations. Firstly, hypertension is known to be a punctual measurement so that the absence of years of hypertension of an individual may be a disadvantage. Secondly, tooth loss may be associated with obesity and diabetes, which are independently associated with hypertension – these variables are likely to be on the causal pathway between tooth loss and hypertension [36, 37]. Therefore, the adjustment in Model 3 might be inappropriate. In addition, although we adjusted for as many potential confounders as possible, residual and unmeasured confounding could not be completely ruled out. Thirdly, it’s better to use inadequate dentition with individuals with fewer than 21 teeth to categorize tooth loss [38]. However, because of our relatively small sample size, there is significant difference in sample size between inadequate and adequate dentition. We will consider using the classification above in our future study with larger sample size. In the fourth place, as a study conducted in urban Shanghai, participants have a higher level of education than other areas in China. Therefore, the study findings may not be generalizable to the wider older population in China. Finally, our findings were based on a cross-sectional study, therefore we were only able to examine the association between tooth loss and hypertension.

## Conclusions

In conclusion, among older Chinese adults, we observed an association between tooth loss and hypertension. Low numbers of remaining teeth might be considered as an impact factor for hypertension among the Chinese older population. Longitudinal and prospective studies are needed to further elucidate the causal association between oral health and hypertension.

## Supplementary information

**Additional file 1.** Oral Health Assessment Questionaire for Adults

## Data Availability

The authors can share their relevant raw data supporting their findings. If any scientist wishes to use them for non-commercial purposes, without breaching participant confidentiality, he or she can contact the authors directly, and they will share their raw data freely with him or her.

## Abbreviations

ANOVA: Analysis of Variance
BMI: Body Mass Index
CI: Confidence Interval
DBP: Diastolic Blood Pressure
OR: Odds Ratio
SAS: The Shanghai Aging Study
SBP: Systolic Blood Pressure
SD: Standard Deviation
