# Supplementary material for: Association between tooth loss and hypertension among older Chinese adults: a community-based study
Source: BMC Oral Health. 2019 Dec 9;19:277. doi: 10.1186/s12903-019-0966-3 (PMC6902561; doi:10.1186/s12903-019-0966-3)
Supplement: Supplementary file 1 — Additional file 1. Oral Health Assessment Questionaire for Adults [file 12903_2019_966_MOESM1_ESM.docx]

**Supplementary file 1**

**Oral Health Assessment Questionnaire for Adults**

| ID: | | Name: | |
| --- | --- | --- | --- |
| Gender: | Occupation: | Nationality: | Residence: |
| Education years: | | Birth date: | |
| Date of assessment: | | | Examiner: |

Oral mucosa lesions

| - 0=absence of condition 1=presence of condition | |
| --- | --- |
| □ | □ |
| □ | □ |
| □ | □ |
| Condition  0 = No abnormal condition  1 = Malignant tumour (oral cancer)  2 = Leukoplakia  3 = Lichen planus  4 = Ulceration (aphthous, herpetic, traumatic)  5= Acute necrotizing ulcerative gingivitis (ANUG)  6 = Candidiasis  7 = Abscess  8= Other condition (specify if possible) 9 = Not recorded | Location  0 = Vermillion border  1 = Commissures  2 = Lips  3 = Sulci  4 = Buccal mucosa  5 = Floor of the mouth  6 = Tongue  7 = Hard and/or soft palate  8 = Alveolar ridges/gingiva  9=Not recorded |

| Self-report dentition status | |
| --- | --- |
| Do you have caries | □Yes, ______number □No |
| Have you ever experienced tooth loss | □Yes, ______number □No |
| Have you ever been diagnosed for periodontitis | □Yes □No |

| Dentition status | | | | | | | | | | | | | | | | |
| --- | --- | --- | --- | --- | --- | --- | --- | --- | --- | --- | --- | --- | --- | --- | --- | --- |
|  | 18 | 17 | 16 | 15 | 14 | 13 | 12 | 11 | 21 | 22 | 23 | 24 | 25 | 26 | 27 | 28 |
| Crown | □ | □ | □ | □ | □ | □ | □ | □ | □ | □ | □ | □ | □ | □ | □ | □ |
| Root | □ | □ | □ | □ | □ | □ | □ | □ | □ | □ | □ | □ | □ | □ | □ | □ |
|  | 48 | 47 | 46 | 45 | 44 | 43 | 42 | 41 | 31 | 32 | 33 | 34 | 35 | 36 | 37 | 38 |
| Crown | □ | □ | □ | □ | □ | □ | □ | □ | □ | □ | □ | □ | □ | □ | □ | □ |
| Root | □ | □ | □ | □ | □ | □ | □ | □ | □ | □ | □ | □ | □ | □ | □ | □ |
| Permanent teeth  Status:  0=Sound  1=Caries  2=Filled w/caries  3=Filled, no caries  4=Missing due to caries  5=Missing for any another reason  6=Fissure sealant  7=Fixed dental prosthesis/crown abutment, veneer, implant  8=Unerupted  9=Not recorded | | | | | | | | | | | | | | | | |

| Periodontal status (CPI modified) | | | | | | | | | | | | | | | | | |
| --- | --- | --- | --- | --- | --- | --- | --- | --- | --- | --- | --- | --- | --- | --- | --- | --- | --- |
|  | 18 | 17 | 16 | 15 | 14 | 13 | 12 | | 11 | 21 | 22 | 23 | 24 | 25 | 26 | 27 | 28 |
| Bleeding | □ | □ | □ | □ | □ | □ | □ | | □ | □ | □ | □ | □ | □ | □ | □ | □ |
| Calculus | □ | □ | □ | □ | □ | □ | □ | | □ | □ | □ | □ | □ | □ | □ | □ | □ |
| Pocket | □ | □ | □ | □ | □ | □ | □ | | □ | □ | □ | □ | □ | □ | □ | □ | □ |
| AL | □ | □ | □ | □ | □ | □ | □ | | □ | □ | □ | □ | □ | □ | □ | □ | □ |
|  | 48 | 47 | 46 | 45 | 44 | 43 | 42 | | 41 | 31 | 32 | 33 | 34 | 35 | 36 | 37 | 38 |
| Bleeding | □ | □ | □ | □ | □ | □ | □ | | □ | □ | □ | □ | □ | □ | □ | □ | □ |
| Calculus | □ | □ | □ | □ | □ | □ | □ | | □ | □ | □ | □ | □ | □ | □ | □ | □ |
| Pocket | □ | □ | □ | □ | □ | □ | □ | | □ | □ | □ | □ | □ | □ | □ | □ | □ |
| AL | □ | □ | □ | □ | □ | □ | □ | | □ | □ | □ | □ | □ | □ | □ | □ | □ |
| Gingival Bleeding  Score  0=Absence of condition  1=Presence of condition  9=Tooth excluded  X=Tooth not present | | | | | | | | Dental calculus  Score  0=Absence of condition  1=Presence of condition  9=Tooth excluded  X=Tooth not present | | | | | | | | | |
| Pocket  Score  0=Absence of condition  1=Pocket 4-5mm  2=Pocket 6mm or more  9=Tooth excluded  X=Tooth not present | | | | | | | | Attachment loss(AL)  Severity  0 = 0-3mm  1 = 4-5mm Cemento-enamel junction(CEJ) within black band  2 = 6-8mm CEJ between upper limit and 8.5mm ring  3 = 9-11mm CEJ between 8.5mm and 11.5mm ring  4 = 12mm or more CEJ beyond 11.5mm ring  X = Excluded sextant  9 = Not recorded | | | | | | | | | |

| Dentures | |
| --- | --- |
| □ | 0=No denture  1=Implant  2=Fixed denture  3=partial denture  4=complete denture  5=Irregular denture  6=Not recorded |
